# Supplementary material for: Dose-Dependent Effects in Plasma Oncotherapy: Critical In Vivo Immune Responses Missed by In Vitro Studies
Source: Biomolecules. 2023 Apr 21;13(4):707. doi: 10.3390/biom13040707 (PMC10136314; doi:10.3390/biom13040707)
Supplement: Supplementary file 1 [file biomolecules-13-00707-s001.zip › biomolecules-2241539-supplementary.pdf]

Supplementary Information

Dose-Dependent Effects in Plasma Oncotherapy: Critical In Vivo Immune Responses Missed by In Vitro Studies

Yuanyuan He [1,2,†], Fanwu Gong [3,†], Tao Jin [1], Qi Liu [1], Haopeng Fang [3], Yan Chen [4], Guomin Wang [5,6], Paul K. Chu [6,7,8], Zhengwei Wu [1,4,*] and Kostya (Ken) Ostrikov [9]

1 School of Nuclear Science and Technology, University of Science and Technology of China, Hefei 230026, China

2 Department of Geriatrics, Division of Life Sciences and Medicine, The First Affiliated Hospital of USTC, University of Science and Technology of China, Hefei 230001, China

3 Department of Medical Oncology, Division of Life Sciences and Medicine, The First Affiliated Hospital of USTC, University of Science and Technology of China, Hefei 230001, China

4 Joint Laboratory of Plasma Application Technology, Institute of Advanced Technology, University of Science and Technology of China, Hefei 230026, China

5 Department of Orthopedics, School of Medicine, Shanghai Tenth People's Hospital, Tongji University, Shanghai 200072, China

6 Department of Physics, City University of Hong Kong, Tat Chee Avenue, Kowloon, Hong Kong 999077, China

7 Department of Materials Science and Engineering, City University of Hong Kong, Tat Chee Avenue, Kowloon, Hong Kong 999077, China

8 Department of Biomedical Engineering, City University of Hong Kong, Tat Chee Avenue, Kowloon, Hong Kong 999077, China

9 School of Chemistry and Physics and QUT Centre for Biomedical Technologies, Queensland University of Technology (QUT), Brisbane, QLD 4000, Australia

* Correspondence: wuzw@ustc.edu.cn

† These authors contributed equally to this work.

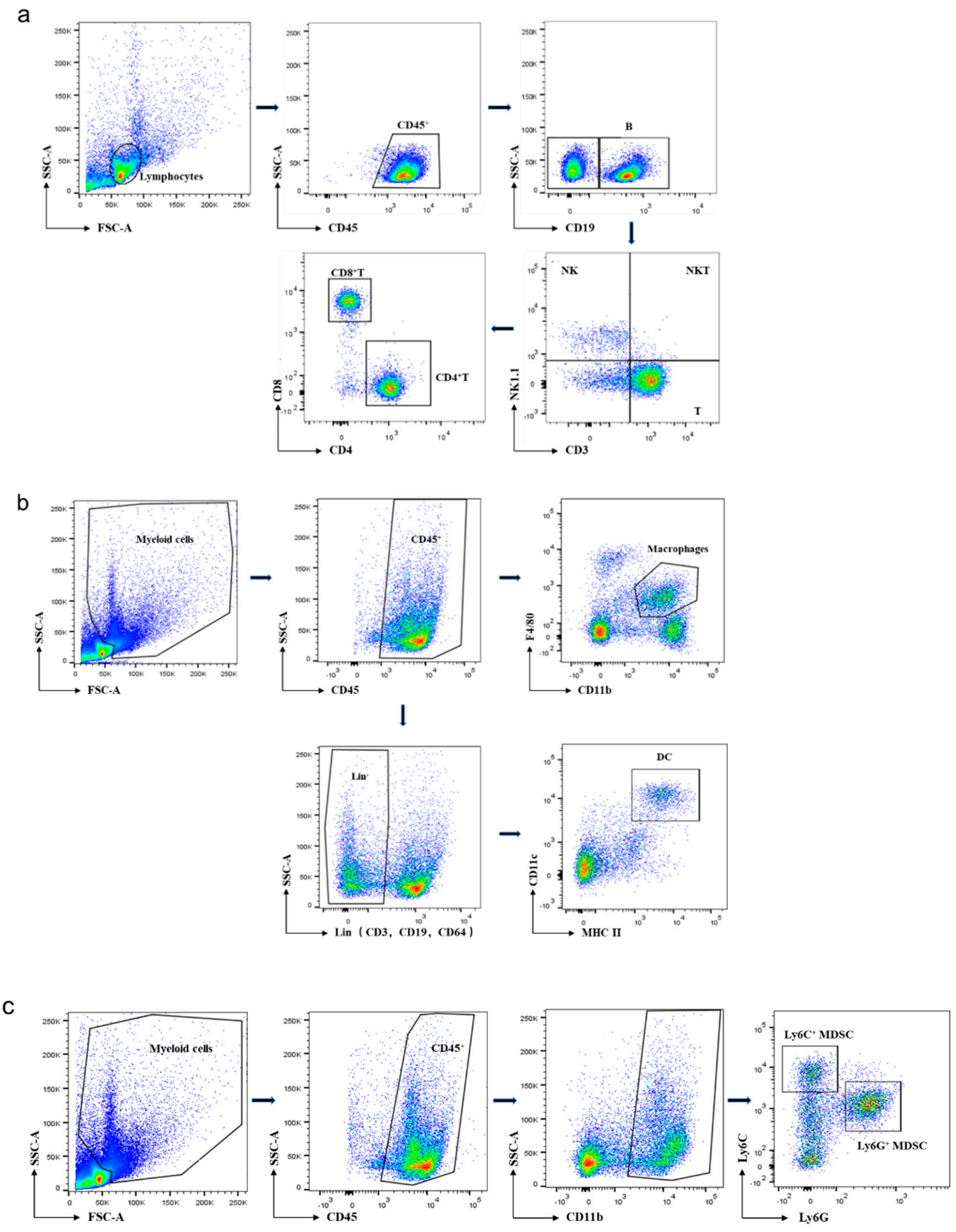

Figure S1. Gating strategies of several immune cell subsets of mice spleen and tumor for flow cytometry analysis: (a) Gating strategies of CD8$^+$T, CD4$^+$T, B, NK, and NKT cell subsets for flow cytometry analysis, n = 5. (b) Gating strategies of CD8$^+$T, CD4$^+$T, B, NK, and NKT cell subsets for flow cytometry analysis, n = 5. In DCs gating, Lin$^-$

(CD3$^-$, CD19$^-$, CD64$^-$) excludes the T, B cell and macrophage subsets. (c) Gating strategies of total MDSC, Ly6G$^+$MDSC, and Ly6C$^+$MDSC subsets for flow cytometry analysis, n = 5. The adhesive cells are removed and the dead cells are stained with DAPI prior to analyzation of the cell surface markers. The data are acquired from 5 biologically independent animals.

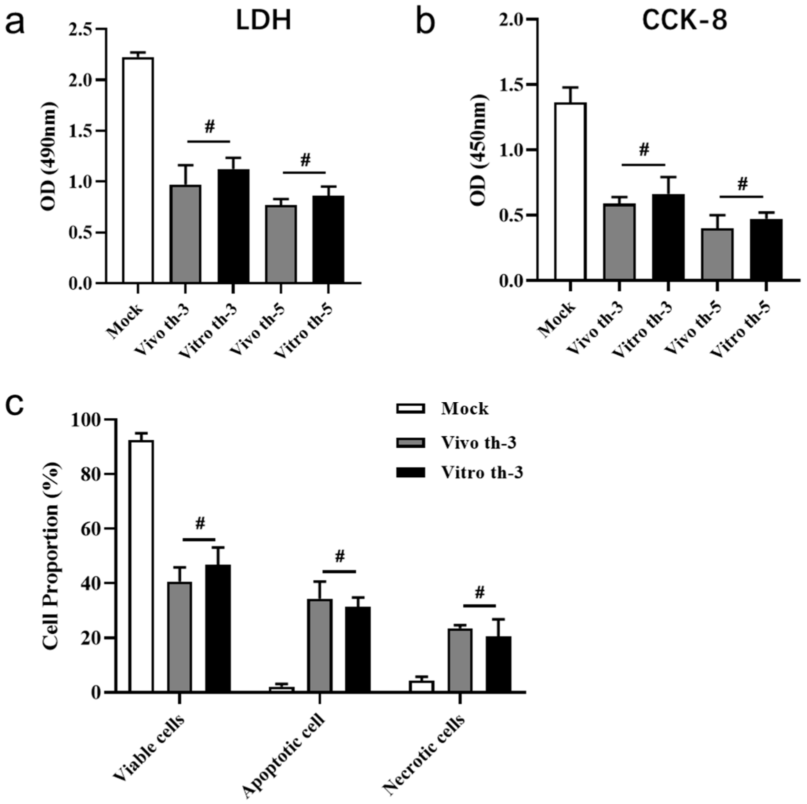

Figure S2. Cytotoxicity and apoptosis of MC38 cells induced by the CAP *in vivo* and *in vitro* devices. The MC38 cells were normally cultured (Mock) or treated with CAP for three days using both the CAP *in vivo* and *in vitro* devices. In the CAP treatment, the MC38 cells were treated by the CAP *in vivo* or *in vitro* devices for 3 or 5 minutes per day for 3 consecutive days. (a, b) LDH and CCK-8 assays of the MC38 cells

suspensions in the control group (Mock) and PT groups, n = 5. (c) Flow cytometry analysis of the apoptotic and necrotic MC38 cells percentages in the Mock and th-3 PT group using Annexin-V/PI staining, n = 3.

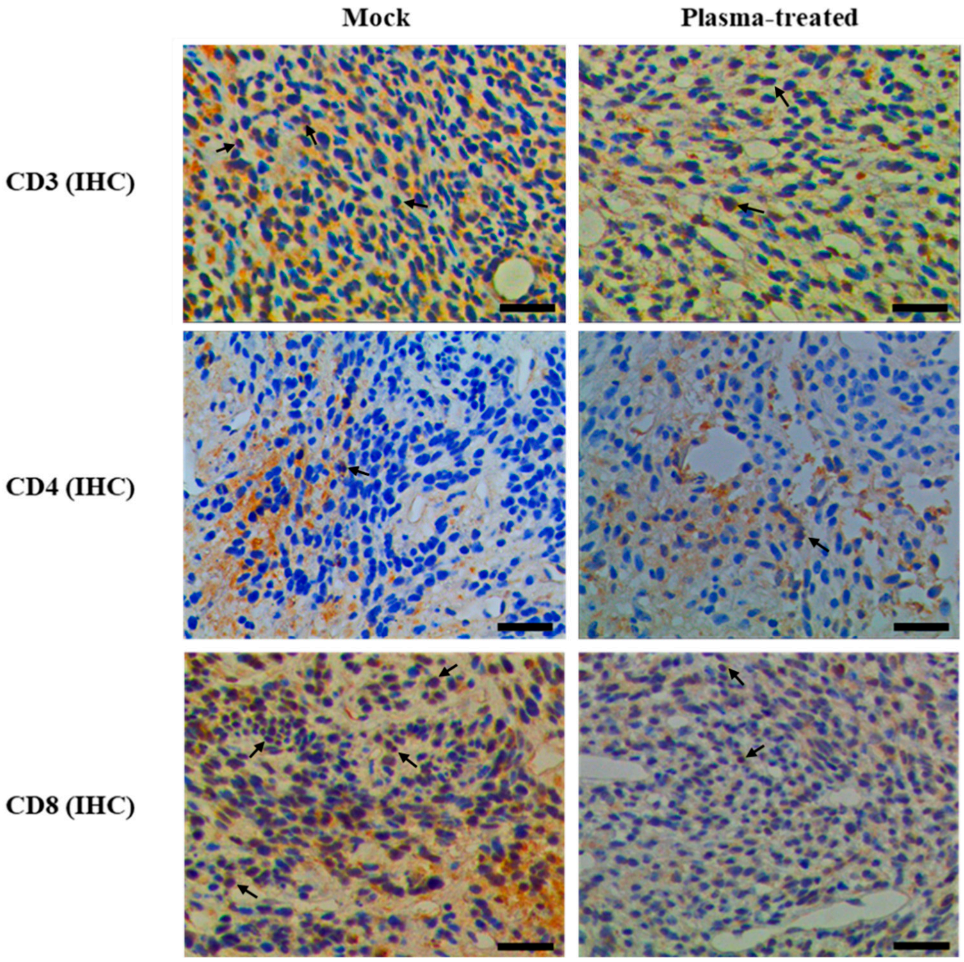

Figure S3. Immunohistochemistry (IHC) staining of CD3, CD4 and CD8 in the tumor tissues of control and PT mice. Immunohistochemical (IHC) staining of CD3, CD4, and CD8 in the serial sections from tumor tissues of the control and PT mice. The arrows indicate the $CD3^+$, $CD4^+$, and $CD8^+$ T cells. Scale bars are 50 μm. The data are acquired from 5 biologically independent animals.

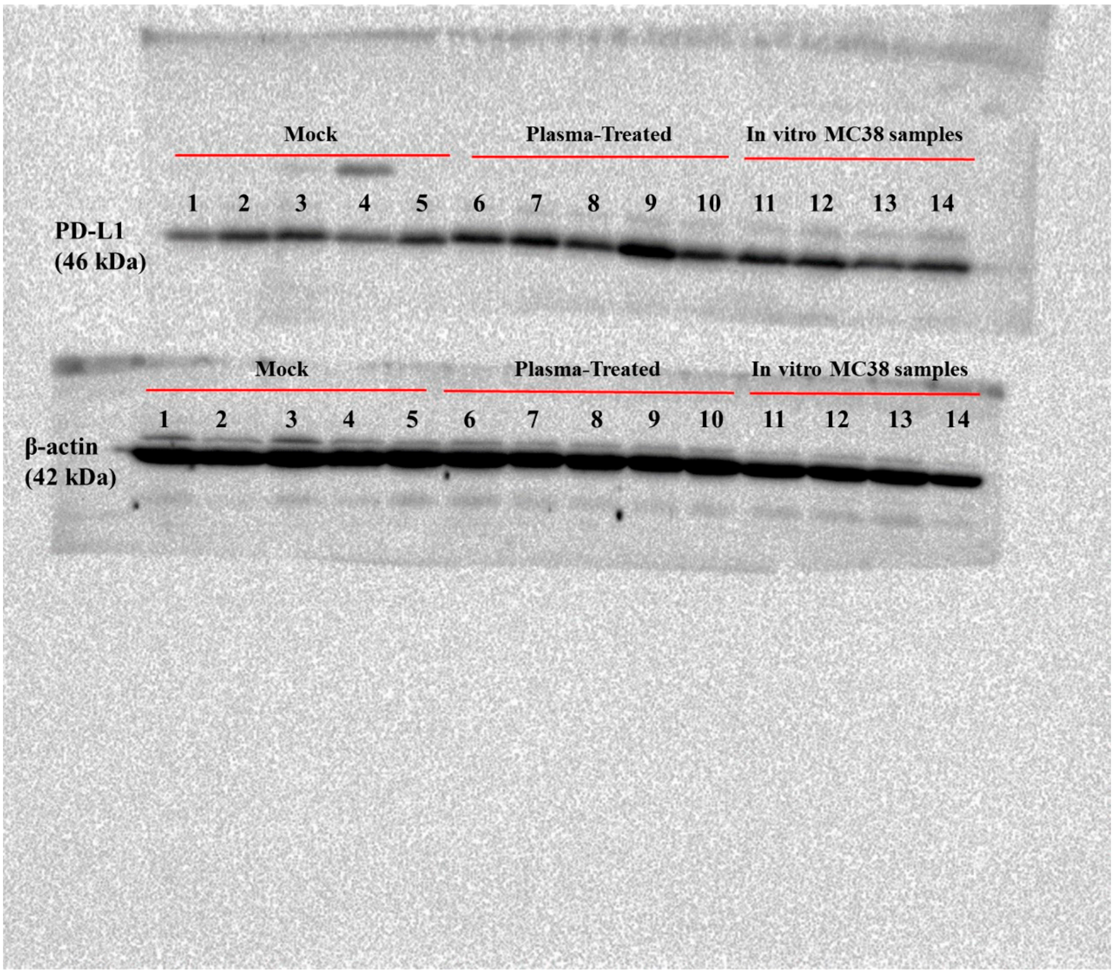

Figure S4. Original image of Figure 5C. Western Immunoblot (WB) analysis to determine the PD-L1 expression in the tumor tissues of the control and PT group mice. Lane 1-5: WB samples of tumor tissues in the control group mice (mock, n=5); Lane 6-10: WB samples of tumor tissues in the PT group mice (Plasma-Treated, n=5); Lane 11-14: WB samples of in vitro MC38 cell samples of control (Lane 11-12) and Th-3 (Lane 13-14) group, not shown in Figure 5C.

Table S1. Primers for the q-PCR analysis in this study.

Genes	Primer sequence(5'to3')	Product lengh（bp）
PD1	F：TGCCCTAGTGGGTATCCCTG	22.00
	R：AAGGCTCCTCCTTCAGAGTG	22.00
PD-L1	F：GTCACTTGCTACGGGCGTTT	22.00
	R：CCCAGTACACCACTAACGCA	22.00
PD-L2	F：GTGCTGGGTGCTGATATTGAC	23.00
	R：AAAATCGCACTCCAGGCTCA	22.00
CTLA-4	F：TGGGTTTTACTCTGCTCCCTG	23.00
	R：GTCACCTGTATGGCTTCAGAG	23.00
CD80	F：TTTCAGACCGGGGCACATAC	22.00
	R：GGGGGTAGAGAAGTCAGCTTT	23.00
CD86	F：GTGATCTTCGGGAATGCTGCT	23.00
	R：CTCCACGGAAACAGCATCTGAG	24.00
Tim3	F：AGACATCAAAGCAGCCAAGGT	23.00
	R：TCCGTGGTTAGGGTTCTTGG	22.00
Galectin	F：GGGGCGCAAACAGAAAACTC	20.00
	R：CTTGGACGGGTAAAGCCCAT	20.00
Lag3	F：GGGGACTTCTCTCTGTGGTTG	21.00
	R：GGACTAGCAATCATCGAGGC	20.00

Table S2. Antibodies for flow cytometry, immunoblot analysis, IHC, and IF assays.

**Antibodies**	**Catalog number**	**Manufacturer**	**Application**	**References (doi)**
CD3-PE	100308	Biolegend	FCM	10.1016/j.vaccine.2014.02.038
CD19-PE	115508	Biolegend	FCM	10.1016/j.immuni.2018.12.011
CD64-PE	558455	BD	FCM	10.4049/jimmunol.170.5.2549
Ly6G-PE	561104	BD	FCM	10.1158/0008-5472
CD69-FITC	104505	Biolegend	FCM	10.1182/blood-2007-07-103200
MHC II-FITC	107605	Biolegend	FCM	10.1084/jem.20101574
Ly6C-FITC	128005	Biolegend	FCM	10.4049/jimmunol.1402210
CD8α-APC	100711	Biolegend	FCM	10.1182/blood-2009-04-214718
F4/80-APC	123115	Biolegend	FCM	10.1038/s41596-021-00644-9
Gr1-APC	108411	Biolegend	FCM	10.1158/0008-5472
CD45-PE-Cy5	103132	Biolegend	FCM	10.1038/s41596-021-00644-9
NK1.1-PE-Cy7	108713	Biolegend	FCM	10.4049/jimmunol.180.12.7818
CD11c-PE-Cy7	117318	Biolegend	FCM	10.1182/blood-2014-04-568956
CD3-APC-Cy7	100330	Biolegend	FCM	10.1016/j.vaccine.2014.02.038
CD11b-APC-Cy7	101226	Biolegend	FCM	10.1038/nm.2159
CD4-BV510	563106	BD	FCM	10.1182/blood-2008-04-153866
PD-1	ab52587	Abcam	IHC	10.1038/s41586-021-03362-0
PD-L1	66248–1-Ig	Proteintech	IHC/IF	10.1016/j.ccell.2020.02.006
CD3	17617-1-AP	Proteintech	IHC	10.1038/s41467-019-11893-4
CD4	67786-1-Ig	Proteintech	IHC	10.3389/fimmu.2022.1042072
CD8α	ab209775	Abcam	IHC	10.1126/sciadv.aax3160
HRP-Goat anti-Rabbit IgG	111-035-003	Jackson Immuno	IHC	10.1126/sciadv.adg1036
CY3-Goat anti-Rabbit IgG	SA00009-2	Proteintech	IF	10.1038/s41586-018-0010-9
PD-L1	66248-1-Ig	Proteintech	WB	10.1016/j.cell.2020.05.028
β-actin	20536-1-AP	Proteintech	WB	10.1038/s41586-022-05499-y
HRP-Goat anti-Mouse IgG	SA00001-1	Proteintech	WB	10.1038/s41592-021-01182-8
